# c-*erb*B-2 is not a major factor in the development of colorectal cancer

**DOI:** 10.1038/sj.bjc.6600127

**Published:** 2002-02-12

**Authors:** J A McKay, J F Loane, V G Ross, M-M Ameyaw, G I Murray, J Cassidy, H L McLeod

**Affiliations:** Department of Medicine and Therapeutics, University of Aberdeen, Institute of Medical Sciences, Foresterhill, Aberdeen AB25 2Z, UK; Department of Pathology, University of Aberdeen, Institute of Medical Sciences, Foresterhill, Aberdeen AB25 2ZD, UK

**Keywords:** colorectal cancer, c-*erb*B-2, immunohistochemistry, PCR–RFLP, polymorphism, prognosis

## Abstract

We have investigated c-*erb*B-2 protein expression in a large cohort of well-characterized colorectal tumours, and in a subset of lymph node metastases. We have also evaluated a Val^655^Ile single nucleotide polymorphism, which is associated with an increased risk of breast cancer, in a subset of the colorectal cancer patients and in healthy control subjects. Immunohistochemical studies revealed that while 81.8% of tumours expressed c-*erb*B-2, in the majority of cases equivalent levels of c-*erb*-B2 were seen in adjacent normal mucosa. Colon tumours were significantly more likely to express c-*erb*B-2 than rectal tumours (*P*=0.015). Only 52.4% of the metastases displayed staining patterns concordant with their primary tumour, indicating that determination of c-*erb*B-2 protein in colorectal tumours cannot predict the status of lymph node metastases. PCR–RFLP analysis of the Val^655^Ile single nucleotide polymorphism demonstrated that allele frequencies were identical between colorectal cancer patients and a control group of Caucasian subjects (Ile=0.80 and Val=0.20 in each case), indicating that it is not related to the risk of developing colorectal cancer in this population. Furthermore, there was no relationship between c-*erb*B-2 protein expression and gene polymorphism (*P*=0.58). In terms of prognosis, no association was seen between either c-*erb*B-2 protein expression or the presence of the Val allele and patient survival (*P*>0.05 in each case), suggesting that c-*erb*B-2 is not a prognostic marker in colorectal cancer.

*British Journal of Cancer* (2002) **86**, 568–573. DOI: 10.1038/sj/bjc/6600127 www.bjcancer.com

© 2002 Cancer Research UK

## 

Colorectal cancer is the second most common malignancy in the developed world (Parkin *et al*, 1993) and despite advances in treatment strategies it remains a major cause of cancer mortality. Currently, prognosis and choice of therapy are based solely on the stage of the disease at presentation, which may not accurately predict disease outcome. Thus, it is widely accepted that additional, novel markers of disease progression need to be identified, in order to individualize therapy accordingly (McLeod and Murray, 1999).

The c-*erb*B-2 (Her2, *neu*) proto-oncogene, located at chromosome 17q21, encodes a 185 kDa glycoprotein which is a member of the type I kinase receptor family, and is involved in mediating a number of normal cellular processes including proliferation (Hung and Lau, 1999). c-*erb*B-2 protein is frequently expressed at low levels in a variety of adult epithelial cells, however aberrant activation of c-*erb*B-2 due to amplification and/or overexpression can contribute to unrestrained proliferation and tumour development or progression (Klapper *et al*, 2000). Clinically, c-*erb*B-2 amplification and/or overexpression has been associated with poor prognosis in a number of tumours, such as breast (Slamon *et al*, 1987; Winstanley *et al*, 1991), ovarian (Slamon *et al*, 1989; Berchuck *et al*, 1990) and gastric (Amadori *et al*, 1997; Yonemura *et al*, 1998) neoplasms. However, the utility of c-*erb*B-2 as a prognostic marker has not yet been established in large cohorts of colorectal tumours.

Further study of c-*erb*B-2 in breast tumours recently demonstrated that a Val^655^Ile single nucleotide polymorphism (SNP) in the transmembrane coding region of the c-*erb*B-2 gene (Papewalis *et al*, 1991) was associated with an increased risk of breast cancer (Xie *et al*, 2000). While it has not yet been determined if this polymorphism affects the ability of c-*erb*B-2 to transform cells, and/or affects its tyrosine kinase activity, the relationship between the Val allele and breast cancer suggests that this polymorphism may be functionally important.

In addition to its roles as a prognostic marker and a risk factor, c-*erb*B-2 is also the target of novel anti-cancer therapies, either in the form of antibody-based therapy or tyrosine kinase inhibitors (Mendelsohn and Baselga, 2000). Therefore, it is essential to accurately assess the frequency of overexpression of c-*erb*B-2 in colorectal tumours compared to normal tissue, and also to evaluate c-*erb*B-2 expression in secondary lesions, in order to gauge the likely efficacy of anti-c-*erb*B-2 therapy in such tumours.

In this study, we have determined the frequency of overexpression of c-*erb*B-2 protein in a large series of well-characterized colorectal tumours, and in a subset of paired lymph node metastases. In addition, we have evaluated the Val^655^Ile SNP in a subgroup of 151 colorectal cancer patients and 257 Caucasian control subjects to determine if there is a relationship between the Val allele and risk of colorectal cancer. Moreover, we have examined c-*erb*B-2 protein overexpression and the presence of the Val allele with respect to clinicopathological information, including patient survival, to evaluate the utility of c-*erb*B-2 as a prognostic marker in colorectal cancer.

## MATERIALS AND METHODS

### Patient information

Archived tumour samples were available from 249 patients who underwent elective surgery for colorectal cancer at Grampian University Hospitals NHS Trust (Scotland, UK). Tumours were collected between 1994 and 1998 at the Department of Pathology, University of Aberdeen, as part of the Aberdeen Colorectal Initiative database. Samples were routinely fixed in 10% neutral buffered formalin for 24 h and embedded in paraffin-wax. An experienced gastrointestinal pathologist confirmed the diagnosis of adenocarcinoma following review of all cases. Detailed clinicopathological data (patient gender, site of primary tumour, degree of differentiation, Dukes' stage and patient age) is available for each sample. Cases of peri-operative death were excluded from survival analysis to avoid events that were not associated with disease. One hundred and sixty-three of the 244 eligible patients were alive at the most recent assessment (October 2000), with a median follow-up of 43 months (range 25–80 months).

A subset of 151 samples was used to determine the allele frequencies of the Val^655^Ile SNP in colorectal cancer. The genotype and allele frequencies of these colorectal cancer samples were compared with those of 257 healthy blood donor controls from Aberdeen, UK, consisting of 140 male and 117 female volunteers, median age 38 years (range, 17–66 years). All studies obtained relevant approval from local ethical committees.

### Immunohistochemistry

c-*erb*B-2 protein expression was analyzed in colorectal tumour samples using immunohistochemistry. An avidin/biotin/horseradish peroxidase development system was used as previously described (McKay *et al*, 2000a,b) Formalin-fixed wax embedded sections (5 μm) were dewaxed, rehydrated and endogenous peroxidase activity blocked using a 3% H_2_O_2_/methanol solution. Antigen retrieval was achieved by microwaving sections for 20 min in 10 mM citrate buffer pH 6.0, and endogenous biotin activity was blocked using a biotin blocking kit (Vector Laboratories Ltd, Peterborough, UK) to prevent non-specific background staining. Following incubation with anti-c-*erb*B-2 monoclonal antibody (NCL-CB11, 1 in 100 dilution; Novocastra, Newcastle upon Tyne, UK), biotinylated rabbit anti-mouse immunoglobulin (Dako A/S Ltd, Glostrup, Denmark) and streptavidin/biotin/ horseradish peroxidase complex (Dako A/S Ltd), sites of bound antibody were visualized using liquid DAB Plus (Dako A/S Ltd). Slides were then counterstained with Mayer's haematoxylin and analyzed using light microscopy. Sections of an invasive breast adenocarcinoma were used as positive controls, and slides were incubated with TBS in place of primary antibody for negative controls.

### Scoring systems

Sections were scored semi-quantitatively according to the following USA FDA-approved scoring system (Jacobs *et al*, 1999): 0, no immunostaining; 1+, complete membranous immunostaining of <10% of tumour cells; 2+, weak complete membranous staining of >10% of tumour cells; 3+, strong complete membranous staining of >10% of tumour cells. Scores of 0 or 1+ indicate a negative tumour, while scores of 2+ and 3+ were regarded as positive expression of c-*erb*B-2. In addition, any normal colonic mucosa present on the section was scored using the same system, and the normal score subtracted from the tumour score. Using this modified system, only cases with a value of 2 were considered c-*erb*B-2 positive as previously described (Jacobs *et al*, 1999). Immunostained tumour sections were analyzed independently by two investigators without prior knowledge of clinicopathological data and discrepancies were resolved by simultaneous re-evaluation.

### DNA extraction

Genomic DNA was extracted from whole blood samples (5 ml) using a sodium perchlorate-chloroform extraction method (Nucleon II kit; Scotlab, Coatbridge, UK). Following extraction, DNA was resuspended in 1 ml TE buffer (10 mM Tris-HCl, 1 mM ethylenediamine tetraacetic acid, pH 8.0) and stored at 4^0^C. Genomic DNA was also extracted from paraffin sections containing formalin-fixed normal colon tissue. Dewaxed and rehydrated material was placed in a microfuge tube, and digested with 0.5 mg ml^−1^ proteinase K for 4 h at 55^0^C. Samples were then heated to 95^0^C for 3 min to inactivate the enzyme, and spun briefly to pellet any cell debris. The resulting supernatant was used directly in PCR analysis.

### PCR–RFLP analysis

Analysis was carried out essentially as previously described (Papewalis *et al*, 1991; Ameyaw *et al*, 2000; Xie *et al*, 2000). Each PCR reaction (50 μl final volume) consisted of 1 μl of sample DNA, 10 mM Tris-HCl, pH 8.3, 50 mM KCl (Perkin Elmer, Cheshire, UK), 1.5 mM MgCl_2_ (Perkin Elmer), 200 μM each of dATP, dTTP, dGTP and dCTP (Promega, Southampton, UK), 1 M betaine, 1.25U AmpliTaq Gold (Perkin Elmer) and 100 ng each of forward and reverse primers (Gibco–BRL, Paisley, UK). Primers used in the analysis of the c-*erb*B-2 Val^655^Ile polymorphism in genomic DNA were 5′-AGA GAG CCA GCC CTC TGA CGT CCA T (forward primer HN-5), and 5′-TCC GTT TCC TGC AGC AGT CTC CGC A (reverse primer HN-6, Papewalis *et al*, 1991). PCR was carried out in a Hybaid Omnigene thermocycler (Hybaid, Middlesex, UK), and consisted of an initial heating at 95°C for 12 min to activate the enzyme, followed by 35 cycles of denaturation at 94°C for 30 s, annealing at 62°C for 30 s and extension at 72°C for 30 s, with a final extension at 72°C for 7 min. Genomic DNA known to be homozygous for the Val allele was included as a positive control, while DNA was omitted from negative control samples.

**Figure 1:**
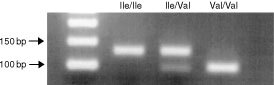
PCR–RFLP analysis of the c-*erb*B-2 Val^655^Ile SNP.

PCR product (10 μl) was digested with 5 U *Bsm*AI (New England BioLabs, Hertfordshire, UK) at 55°C for 2 h, and visualized by electrophoresis on 2.5% agarose containing 0.5 μg ml^−1^ ethidium bromide. The 148 bp PCR product generated was cut by *Bsm*AI into fragments of 122 and 26 bp if the Ile allele was present, whereas the product from the Val allele was cut to produce fragments of 90, 32 and 26 bp ( Figure 1

). Genotypes were assigned following visual identification of unequivocal bands on gels where complete digestion of the homozygous Val positive control DNA had occurred.

### Statistics

The relationship between c-*erb*B-2 protein expression in primary colorectal tumours and lymph node metastases was evaluated using the kappa test. Expression of c-*erb*B-2 protein, and the Val^655^Ile SNP, were assessed with respect to Dukes' stage, degree of differentiation, site of the primary tumour and patient gender using the chi-squared test, while patient age was examined using the Mann–Whitney *U*-test. The association between c-*erb*B-2 protein expression and the Val^655^Ile SNP was examined using the chi-squared test. The effects of c-*erb*B-2 on survival were tested using Kaplan–Meier survival plots and analyzed using the log rank test. Significance levels were set at *P*<0.05, and all statistical analyzes were carried out using SPSS for Windows 95 version 9.0 (SPSS UK Ltd, Woking, Surrey, UK).

## RESULTS

### Immunohistochemistry

#### Primary colorectal tumours

The expression of c-*erb*B-2 protein was examined in 249 colorectal tumours. One primary tumour gave inconsistent immunostaining results on repeated evaluations, and was excluded from further analysis. The distribution of immunostaining in primary colorectal tumours is detailed in Table 1

**Table 1:**
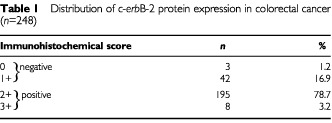
Distribution of c-*erb*B-2 protein expression in colorectal cancer (*n*=248)

, which shows that although 81.5% of tumours expressed c-*erb*B-2, only 8 out of 248 (3.2%) displayed strong immunoreactivity in the membrane of tumour cells ( Figure 2

**Figure 2:**
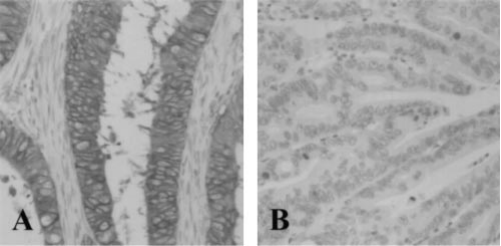
Representative examples of (**A**) strong membranous staining of tumour cells, and (**B**) negative immunoreactivity for c-*erb*B-2, in primary colorectal adenocarcinomas.

). Of the 204 samples where normal colon mucosa could also be assessed, 174 had c-*erb*B-2 scores of 2+ (165 cases) or 3+ (9 cases). Only 24 out of 204 (11.8%) samples displayed higher levels of c-*erb*B-2 in the tumour compared to normal tissue, and none of these samples reached a difference value of ⩾2 (which was the definition of positive c-*erb*B-2 expression using the modified scoring system; Jacobs *et al*, 1999).

#### Lymph node metastases

c-*erb*B-2 expression was also assessed in 42 lymph node metastases from a subgroup of patients with Dukes' stage C or D tumours. Twenty-one (50.0%) of the examined metastases displayed positive c-*erb*B-2 immunoreactivity ( Table 2

**Table 2:**
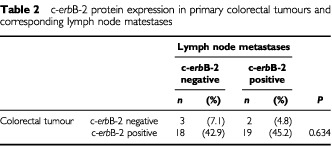
c-*erb*B-2 protein expression in primary colorectal tumours and corresponding lymph node matestases

). Of the 42 paired samples evaluated, 22 out of 42 (52.4%) displayed equivalent c-*erb*B-2 expression in both the primary tumour and the metastatic deposit. Of the remaining 20 pairs, 18 (42.9%) had higher c-*erb*B-2 expression in the primary tumour, while two (4.8%) had elevated c-*erb*B-2 levels in the lymph node lesion. Using the kappa test, which is a measure of agreement between paired samples, only a poor level of concordance was observed (κ=0.048, *P*=0.634; Table 2).

### c-*erb*B-2 protein and clinicopathological information

The expression of c-*erb*B-2 protein was evaluated with respect to patient clinicopathological data, and the results detailed in Table 3

**Table 3:**
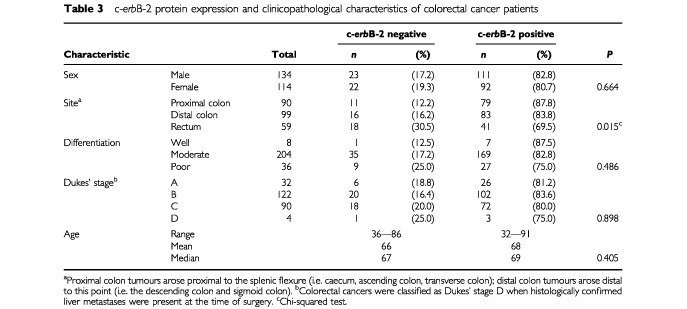
c-*erb*B-2 protein expression and clinicopathological characteristics of colorectal cancer patients

. c-*erb*B-2 expression was associated with the site of the primary tumour, with the frequency of positive c-*erb*B-2 expression decreasing from proximal colon to distal colon to rectum (*P*=0.015). There was no correlation between c-*erb*B-2 protein expression and patient gender, degree of differentiation, tumour stage, or patient age (*P*>0.05 in each case).

### Val^655^Ile SNP in colorectal cancer and control populations

The frequency of a polymorphic Val allele at position 655 of the c-*erb*B-2 gene was assessed in a subset of 151 colorectal cancer patients and in 257 Caucasian control subjects using PCR–RFLP analysis. The genotype and allele frequencies are shown in Table 4

**Table 4:**
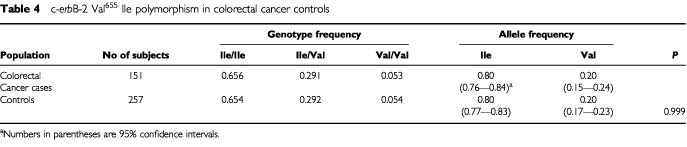
c-*erb*B-2 Val^655^ Ile polymorphism in colorectal cancer controls

. The genotype frequencies were not significantly different from the Hardy–Weinberg equilibrium in either population. These two populations demonstrated an identical Val allele frequency (0.20, *P*=0.999; Table 4).

There was no association between the Val allele and clinicopathological data (i.e. Dukes' stage, differentiation grade, site, gender or age), nor was there a relationship between this polymorphism and c-*erb*B-2 protein expression (*P*=0.582; Table 5

**Table 5:**
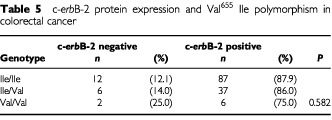
c-*erb*B-2 protein expression and Val^655^ Ile polymorphism in colorectal cancer

).

### c-*erb*B-2 and survival

**Figure 3:**
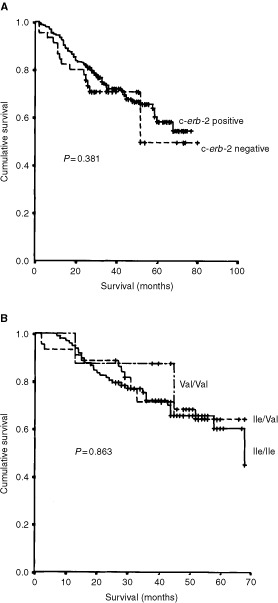
Kaplan–Meier plots of the effects on colorectal cancer patient outcome of (**A**) c-*erb*B-2 protein expression, and (**B**) the Val^655^Ile SNP. Neither of these factors were found to influence survival (*P*>0.05 in each case).

The effects of c-*erb*B-2 protein expression, and the Val^655^Ile SNP, on patient survival were examined. No association was seen either between protein expression, or the presence of the Val allele, and patient outcome (*P*>0.05 in each case; Figure 3

).

## DISCUSSION

The results of the present study indicate that: (1) although c-*erb*B-2 protein expression is frequently observed in colorectal tumours, it is rarely at a higher level than adjacent normal tissue; (2) the expression of c-*erb*B-2 protein in primary colorectal tumours does not accurately reflect the c-*erb*B-2 status of lymph node metastases; (3) a SNP at position 655 of the c-*erb*B-2 gene is not associated with risk of colorectal cancer, nor does it influence protein expression; and (4) c-*erb*B-2 protein status and Val^655^Ile SNP do not influence patient survival in colorectal cancer patients.

The lack of differential expression between colorectal tumour and normal tissue, and the lack of correlation between c-*erb*B-2 protein expression and tumour stage or grade, suggest that c-*erb*B-2 does not play a major role in the development of colorectal adenocarcinoma. There have been previous reports that have associated c-*erb*B-2 protein expression with more aggressive colorectal tumours. For example, colorectal tumours which subsequently developed liver metastases more frequently expressed c-*erb*B-2 protein than those which did not metastasise in an 8-year period (Yang *et al*, 1997), and in a further study cytoplasmic c-*erb*B-2 protein expression was associated with Dukes' stage (Osako *et al*, 1998). However, Berney *et al* (1999) found that c-*erb*B-2 protein was not associated with the risk of developing liver metastases. The present study also found that c-*erb*B-2 protein expression did not have predictive power for patient survival or other features associated with an unfavourable outcome. There are several possible reasons for discrepancies between studies. The small sample size of many studies of c-*erb*B-2 in colorectal cancer provides a source of type II statistical error. The disparate scoring systems used to classify c-*erb*B-2 overexpression (i.e. membranous and/or cytoplasmic immunostaining, per cent of positive tumour cells and/or intensity of immunoreactivity) also make comparison between studies very challenging. In addition, the source of the primary antibody used, the immunohistochemical protocol and the size of the sample set may all lead to inconsistencies between reports. This highlights the need for a standardized system of c-*erb*B-2 classification as previously documented (van de Vijver, 2001).

In addition to its possible role in tumour development, c-*erb*B-2 overexpression is also a target of novel anti-cancer therapies (Sliwkowski *et al*, 1999; Mendelsohn and Baselga, 2000). While such treatments are generally targeted against secondary lesions (McLeod *et al*, 2000), markers of response are usually assessed in primary tumour tissue, and it has been shown that metastatic deposits often display a different biological composition to their primary progenitor tumour cells (McKay *et al*, 2000b; McLeod *et al*, 2000). Such differences may affect the expected efficacy of treatment.

We found that only approximately half of the examined paired samples (52.4%) showed concordance between primary and secondary tumours. While Sun *et al* (1994) found a significant association between c-*erb*B-2 protein expression in colorectal adenocarcinomas and lymph node metastases, 30% of their samples did not demonstrate equivalent immunostaining in primary and secondary tumours. Taken together, these results indicate that the c-*erb*B-2 protein status of colorectal tumours does not accurately reflect the situation in metastatic lesions, and would be unable to predict the likely efficacy of anti-c-*erb*B-2 therapy in a large proportion of secondary tumours.

The recently reported association between a c-*erb*B-2 SNP and risk of breast cancer in a Chinese population (Xie *et al*, 2000), led us to investigate possible links between this SNP and the development of colorectal cancer. We found that the allele frequencies for the Val allele were identical in a subset of our colorectal cancer patients and a control group of healthy Caucasian blood donors. This indicates that the presence of the polymorphic Val allele is not associated with risk of colorectal cancer in Scottish Caucasians. In addition, there was no relationship between the Val allele and c-*erb*B-2 protein immunostaining in colorectal adenocarcinomas. This does not infer a lack of functional significance of the Val allele in other normal or tumour tissues, especially breast malignancy.

Interestingly, the Val allele frequency (0.20) in this study is higher than both the control and case Val allele frequencies (0.11 and 0.15 respectively) seen in Chinese populations (Xie *et al*, 2000). Further reports on the c-*erb*B-2 SNP have also indicated differences in allele frequencies between ethnic groups (Ameyaw *et al*, 2000; Baxter and Campbell, 2001), which is likely to influence the utility of this SNP in global risk analysis.

To date, studies on the prognostic utility of c-*erb*B-2 protein overexpression in colorectal tumours have reached conflicting conclusions. Osako *et al* (1998) found that cytoplasmic c-*erb*B-2 protein was significantly associated with poor prognosis in a group of 146 colorectal tumours, and Kapitanovic *et al* (1997) found that intensity of c-*erb*B-2 staining (membranous and/or cytoplasmic staining) was independently related to survival in 155 colorectal tumours. However, in agreement with our findings, Sun *et al* (1995) showed that membranous c-*erb*B-2 expression in 293 colorectal tumours was not associated with prognosis, while Chamberlain *et al* (1999), found no association between survival and c-*erb*B-2 staining intensity (membranous and/or cytoplasmic staining) in 96 colorectal tumours. As mentioned previously, these discrepancies are likely due to procedural disparities. The present study, which was carried out using highly reproducible automated immunostaining on a large series of colorectal tumours with a minimum of 25 months follow-up, has used recommended guidelines for assessing c-*erb*B-2 overexpression (Jacobs *et al*, 1999), to allow straightforward comparisons with future studies.

There have been no previous reports on the influence of the Val^655^Ile SNP on the clinical outcome of colorectal cancer patients. In the group of patients evaluated in this study, there was no association between the presence of the Val allele and patient survival. In addition, we found no relationship between the c-*erb*B-2 SNP and protein expression, indicating that evaluation of this SNP would be of little benefit in colorectal adenocarcinoma.

In conclusion, while c-*erb*B-2 appears to be highly important in the development and progression of a number of epithelial malignancies, the measurement of its protein expression and evaluation of the Val^655^Ile SNP is unlikely to be of clinical benefit to patients with colorectal adenocarcinoma.
